# Supplementary material for: Distinct trans-placental effects of maternal immune activation by TLR3 and TLR7 agonists: implications for schizophrenia risk
Source: Sci Rep. 2021 Dec 13;11:23841. doi: 10.1038/s41598-021-03216-9 (PMC8668921; doi:10.1038/s41598-021-03216-9)
Supplement: Supplementary file 1 — Supplementary Information. [file 41598_2021_3216_MOESM1_ESM.pdf]

Distinct trans-placental effects of maternal immune activation by TLR3 and TLR7 agonists: implications for schizophrenia risk

Jaedeok Kwon, Maria Suessmilch, Alison McColl, Jonathan Cavanagh, Brian J. Morris

Supplementary Information

Supplementary Table 1. Details of statistical analysis output, related to Figures 1-4 and Supplementary figure1

**Figure (Protein/*gene*)**	**Source**	**F and P values**	**Post-hoc comparisons**
Fig 1 A(IL-6)	Mother injection	F(2,10)=27.61, p=0.000	Poly I:C vs. PBS (p=0.020)<br>Resiquimod vs. PBS (p=0.000)<br>Resiquimod vs. poly I:C (p=0.001)
Fig 1 (TNF-α)	Mother injection	F(2,10)=26.79, p=0.000	Poly I:C vs. PBS (p=0.037)<br>Resiquimod vs. PBS (p=0.000)<br>Resiquimod vs. poly I:C (p=0.001)
Fig 1 C(IL-10)	Mother injection	F(2,10)=57.42, p=0.000	Poly I:C vs. PBS (p=0.012)<br>Resiquimod vs. PBS (p=0.000)<br>Resiquimod vs. poly I:C (p=0.000)
Fig 1 D(CCL2)	Mother injection	F(2,9)=8.50, p=0.013	Poly I:C vs. PBS (p=0.036)<br>Resiquimod vs. PBS (p=0.005)<br>Resiquimod vs. poly I:C (p=0.116)
Fig 1 E(CCL5)	Mother injection	F(2,9)=7.93, p=0.016	Poly I:C vs. PBS (p=0.036)<br>Resiquimod vs. PBS (p=0.007)<br>Resiquimod vs. poly I:C (p=0.132)
Fig 1 F(CCL11)	Mother injection	F(2,10)=23.09, p=0.000	Poly I:C vs. PBS (p=0.090)<br>Resiquimod vs. PBS (p=0.000)<br>Resiquimod vs. poly I:C (p=0.001)
Fig 1 G(CXCL1)	Mother injection	F(2,9)=17.81, p=0.002	Poly I:C vs. PBS (p=0.524)<br>Resiquimod vs. PBS (p=0.001)<br>Resiquimod vs. poly I:C (p=0.001)
Fig 1 H(CXCL10)	Mother injection	F(2,10)=10.12, p=0.006	Poly I:C vs. PBS (p=0.010)<br>Resiquimod vs. PBS (p=0.003)<br>Resiquimod vs. poly I:C (p=0.286)
Fig 1 I(LIF)	Mother injection	F(2,9)=91.63, p=0.000	Poly I:C vs. PBS (p=0.001)<br>Resiquimod vs. PBS (p=0.000)

			Resiquimod vs. poly I:C (p=0.000)
Fig 2 A(*Il-6*)	Mother injection	F(2,78)=73.86, p=0.000	Poly I:C vs. PBS (p=0.053)<br>Resiquimod vs. PBS (p=0.000)<br>Resiquimod vs. poly I:C (p=0.000)
	Mother ID coding (MIA injection)	F(9,78)=3.22, p=0.002	
Fig 2 B(*Tnf-α)*	Mother injection	F(2,78)=46.38, p=0.000	Poly I:C vs. PBS (p=0.519)<br>Resiquimod vs. PBS (p=0.000)<br>Resiquimod vs. poly I:C (p=0.000)
	Mother ID coding (MIA injection)	F(9,78)=1.73, p=0.097	
Fig 2 C(*Il-10*)	Mother injection	F(2,78)=69.85, p=0.000	Poly I:C vs. PBS (p=0.019)<br>Resiquimod vs. PBS (p=0.000)<br>Resiquimod vs. poly I:C (p=0.000)
	Mother ID coding (MIA injection)	F(9,78)=2.12, p=0.038	
Fig 2 D(*Ccl2*)	Mother injection	F(2,78)=2.07, p=0.133	Poly I:C vs. PBS (p=0.245)<br>Resiquimod vs. PBS (p=0.136)<br>Resiquimod vs. poly I:C (p=0.949)
	Mother ID coding (MIA injection)	F(9,78)=1.36, p=0.219	
Fig 2 E(*Ccl5*)	Mother injection	F(2,78)=30.83, p=0.000	Poly I:C vs. PBS (p=0.466)<br>Resiquimod vs. PBS (p=0.000)<br>Resiquimod vs. poly I:C (p=0.000)
	Mother ID coding (MIA injection)	F(9,78)=2.88, p=0.005	
Fig 2F(*Ccl11*)	Mother injection	F(2,78)=33.23, p=0.000	Poly I:C vs. PBS (p=0.417)<br>Resiquimod vs. PBS (p=0.000)<br>Resiquimod vs. poly I:C (p=0.000)
	Mother ID coding (MIA injection)	F(9,78)=1.29, p=0.257	
Fig 2 (*Cxcl1*)	Mother injection	F(2,78)=33.24, p=0.000	Poly I:C vs. PBS (p=0.984)<br>Resiquimod vs. PBS (p=0.000)

			Resiquimod vs. poly I:C (p=0.000)
	Mother ID coding (MIA injection)	F(9,78)=2.81, p=0.007	
Fig 2 H(*Cxcl10*)	Mother injection	F(2,78)=225.77, p=0.000	Poly I:C vs. PBS (p=0.000) Resiquimod vs. PBS (p=0.000) Resiquimod vs. poly I:C (p=0.000)
	Mother ID coding (MIA injection)	F(9,78)=13.31, p=0.000	
Fig 2 I(*Cxcl12*)	Mother injection	F(2,78)=2.93, p=0.059	Poly I:C vs. PBS (p=0.999) Resiquimod vs. PBS (p=0.112) Resiquimod vs. poly I:C (p=0.096)
	Mother ID coding (MIA injection)	F(2,78)=5.37, p=0.000	
Fig 3 A(*Il-6*)	Mother injection	F(2,36)=205.80, p=0.000	Poly I:C vs. PBS (p=0.737) Resiquimod vs. PBS (p=0.000) Resiquimod vs. poly I:C (p=0.000)
	Mother ID coding (MIA injection)	F(9,36)=1.71, p=0.122	
Fig 3 B(*Tnf-α*)	Mother injection	F(2,36)=897.83, p=0.000	Poly I:C vs. PBS (p=0.247) Resiquimod vs. PBS (p=0.000) Resiquimod vs. poly I:C (p=0.000)
	Mother ID coding (MIA injection)	F(9,36)=0.75, p=0.664	
Fig 3 C(*Il-10)*	Mother injection	F(2,34)=65.17, p=0.000	Poly I:C vs. PBS (p=0.121) Resiquimod vs. PBS (p=0.000) Resiquimod vs. poly I:C (p=0.000)
	Mother ID coding (MIA injection)	F(9,34)=2.06, p=0.062	
Fig 3 D(*Ccl2*)	Mother injection	F(2,36)=419.15, p=0.000	Poly I:C vs. PBS (p=0.888) Resiquimod vs. PBS (p=0.000) Resiquimod vs. poly I:C (p=0.000
	Mother ID coding (MIA injection)	F(9,36)=2.92, p=0.011	

Fig 3 E(*Ccl5*)	Mother injection	F(2,36)=635.87, p=0.000	Poly I:C vs. PBS (p=0.092)<br>Resiquimod vs. PBS (p=0.000)<br>Resiquimod vs. poly I:C (p=0.000)
	Mother ID coding (MIA injection)	F(9,36)=3.93, p=0.001	
Fig 3 F(*Ccl11*)	Mother injection	F(2,34)=36.10, p=0.000	Poly I:C vs. PBS (p=0.689)<br>Resiquimod vs. PBS (p=0.000)<br>Resiquimod vs. poly I:C (p=0.000)
	Mother ID coding (MIA injection)	F(9,34)=0.57, p=0.811	
Fig 3 G(*Cxcl1*)	Mother injection	F(2,36)=54.05, p=0.000	Poly I:C vs. PBS (p=0.475)<br>Resiquimod vs. PBS (p=0.000)<br>Resiquimod vs. poly I:C (p=0.000)
	Mother ID coding (MIA injection)	F(9,36)=4.14, p=0.001	
Fig 3 H(*Cxcl10*)	Mother injection	F(2,36)=907.89, p=0.000	Poly I:C vs. PBS (p=0.999)<br>Resiquimod vs. PBS (p=0.000)<br>Resiquimod vs. poly I:C (p=0.000)
	Mother ID coding (MIA injection)	F(9,36)=10.70, p=0.000	
Fig 3 I(*Cxcl12*)	Mother injection	F(2,36)=3.43, p=0.043	Poly I:C vs. PBS (p=0.034)<br>Resiquimod vs. PBS (p=0.408)<br>Resiquimod vs. poly I:C (p=0.312)
	Mother ID coding (MIA injection)	F(9,36)=2.60, p=0.020	
Fig 4 A(*Aif1*)	Mother injection	F(2,36)=18.05, p=0.000	Poly I:C vs. PBS (p=0.469)<br>Resiquimod vs. PBS (p=0.001)<br>Resiquimod vs. poly I:C (p=0.000)
	Mother ID coding (MIA injection)	F(9,36)=4.96, p=0.000	
Fig 4 B(*Cx3cr1*)	Mother injection	F(2,36)=117.14, p=0.000	Poly I:C vs. PBS (p=0.208)<br>Resiquimod vs. PBS (p=0.000)<br>Resiquimod vs. poly I:C (p=0.000)

	Mother ID coding (MIA injection)	F(9,36)=2.91, p=0.011	
Fig 4 C(*Tmem119*)	Mother injection	F(2,36)=11.25, p=0.000	Poly I:C vs. PBS (p=0.000)<br>Resiquimod vs. PBS (p=0.008)<br>Resiquimod vs. poly I:C (p=0.200)
	Mother ID coding (MIA injection)	F(9,36)=4.49, p=0.001	
Fig 4 D(*Ccr2*)	Mother injection	F(2,36)=19.89, p=0.000	Poly I:C vs. PBS (p=0.008)<br>Resiquimod vs. PBS (p=0.000)<br>Resiquimod vs. poly I:C (p=0.009)
	Mother ID coding (MIA injection)	F(9,36)=5.47, p=0.000	
Fig 4 E(*Ly6c2*)	Mother injection	F(2,33)=1.20, p=0.315	Poly I:C vs. PBS (p=0.956)<br>Resiquimod vs. PBS (p=0.348)<br>Resiquimod vs. poly I:C (p=0.461)
	Mother ID coding (MIA injection)	F(9,33)=3.43, p=0.004	
Supplementary Figure1A(*Bdnf)*	Mother injection	F(2,36)=1.96, p=0.156	Poly I:C vs. PBS (p=0.181)<br>Resiquimod vs. PBS (p=0.923)<br>Resiquimod vs. poly I:C (p=0.268)
	Mother ID coding (MIA injection)	F(9,36)=6.18, p=0.000	
Supplementary Figure1B(*Bdnf)*	Mother injection	F(2,78)=3.87, p=0.025	Poly I:C vs. PBS (p=0.119)<br>Resiquimod vs. PBS (p=0.022)<br>Resiquimod vs. poly I:C (p=0.746)
	Mother ID coding (MIA injection)	F(9,78)=1.62, p=0.124	

Supplementary Table 2. Primer sequences

Target	Forward primers	Reverse primers	STD forward primers	STD reverse primers	Tissue used for generating STD
Gapdh	AAT GTG TCC GTC GTG GAT CT	AGA CAA CCT GGT CCT CAG TG	GCA TTG TGG AAG GGC TCA TG	GGC ATC GAA GGT GGA AGA GT	Whole mouse Brain
Tbp	TGC TGT TGG TGA TTG TTG GT	AAC TGG CTT GTG TGG GAA AG	GAG TTG CTT GCT CTG TGC TG	ATA CTG GGA AGG CGG AAT GT	Whole mouse Brain
Il-6	CGG CCT TCC CTA CTT CAC AA	TCA TTT CCA CGA TTT CCC AGA GA	GGA GCC CAC CAA GAA CGA T	TGG TCC TTA GCC ACT CCT TCT	Whole mouse Brain
Tnf-α	CAC CAC CAT CAA GGA CTC AA	GAG GCA ACC TGA CCA CTC TC	TCT GTG AAG GGA ATG GGT GT	GGC TGG CTC TGT GAG GAA	Whole mouse Brain
Il-10	CAG AGA AGC ATG GCC CAG AA	GCT CCA CTG CCT TGC TCT TA	TGC TAA CCG ACT CCT TAA TGC A	GGC CTT GTA GAC ACC TTG GT	Whole mouse Brain
Ccl2	CTC ACC TGC TGC TAC TCA TTC A	CCA TTC CTT CTT GGG GTC A	CAC CAG CAC CAG CCA ACT	GCA TCA CAG TCC GAG TCA CA	Whole mouse Brain
Ccl5	CTG CTG CTT TGC CTA CCT CT	ACA CAC TTG GCG GTT CCT T	CCC TCA CCA TCA TCC TCA CT	TCA GAA TCA AGA GGC CCT CTA TCC	Whole mouse Brain
Ccl11	GCT CAC CCA GGC TCC ATC	TCT CTT TGC CCA ACC TGG TC	CCA CCC ACT CTG CTC CCT AT	GGC ATC CTG GAC CCA CTT C	Mouse liver
Cxcl1	TGC ACC CAA ACC GAA GTC AT	TGG GGA CAC CTT TTA GCA TCT	ACA CTC CAA CAC AGC ACC AT	AAA CAC AGC CTC CCA CAC AT	Whole mouse Brain
Cxcl10	GCT CAA GTG GCT GGG ATG	GAG GAC AAG GAG GGT GTG G	CGA TGG ATG GAC AGC AGA GAG CCT	GAC AAG GAG GGT GTG GGG AGC A	Mouse spleen
Cxcl12	TGC ATC AGT GAC GGT AAA CCA	GCG ATG TGG CTC TCG AAG AA	GTC CTC TTG CTG TCC AGC TCT	CTT CAG CCG TGC AAC AAT CTG	Mouse spleen
Aif1	CTG CCA GCC TAA GAC AAC CA	ATC CCC TCC AGC CTC TCT TC	GTG TGA GAA CGG TCC CAG AA	GAC TCT GGC TCA CGA CTG TT	Whole mouse Brain
Cx3cr1	GCT CAC GAC TGC CTT CTT CT	TGC ACT GTC CGG TTG TTC AT	CGT TCG GTC TGG TGG GAA AT	CCC ATC TCC CTC GCT TGT G	Whole mouse Brain
Tmem119	TGC ACC CCA GGA AAC ATC TC	AGT GGT GCG TTA GGG TGA AG	GAG GGA GCA AAG CCT GTG AA	GCA GAG TGA CAG CGA CAT TG	Whole mouse Brain
Ccr2	CTT GGA ATG ACA CAC TGC TGC	AGC TCA CTC GAT CTG CTG TC	CCA CAC CCT GTT TCG CTG TA	GCC TCA TGC CCT CCT TTC TT	Whole mouse Brain
Ly6c2	ACC CGT CAG TGC CTT TCT TT	CAG TGG GAA CTG CTG CAT TG	GCC TGC AAC CTT GTC TGA GA	TGG ACT CAA CAG GGG TCA TTG	Mouse Hippocampus
Xist	TCC TCG GTT CCA CAC ATT GC	AGA GCA TCC CTC TGC TTT CC	GGC ATG CTT GGT AGA GTG GA	GGA GCC AAG TGA AGG CCT AA	Mouse spleen
Bdnf	CAC TCC ACT GCC CAT GAT GT	GGA GGA GGG AGG GAA AGA GT	TCA AGG TGC TGT TGT CAT TGC	TCC CCA CCT CCA TCC TAG AC	Whole mouse Brain

Supplementary Figures

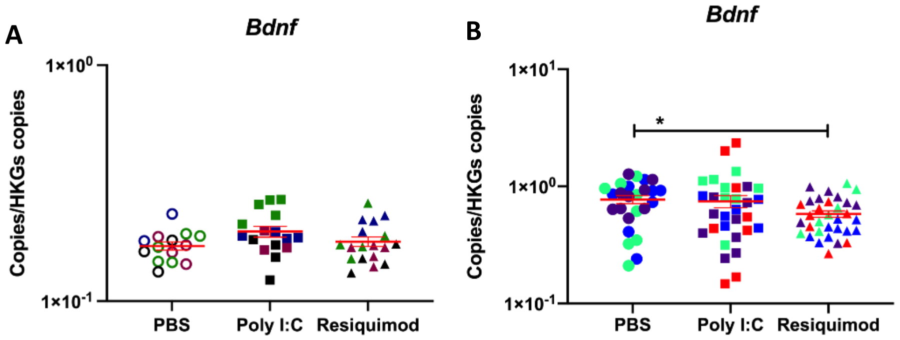

Supplementary Figure 1. Bdnf

Foetal brain (left) and placenta (right)

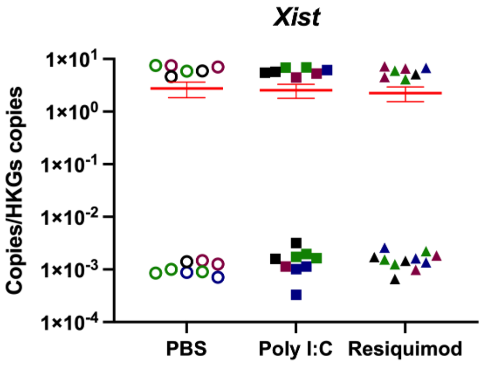

Supplementary Figure 2. A gene for sex determination in E.Brain by RT-qPCR
